# Psychometric Properties of the Chinese Version of Short-Form Community Attitudes Toward Mentally Illness Scale in Medical Students and Primary Healthcare Workers

**DOI:** 10.3389/fpsyt.2020.00337

**Published:** 2020-04-24

**Authors:** Yan Tong, Zhizhong Wang, Yan Sun, Shulan Li

**Affiliations:** ^1^Department of Social Medicine, School of Public Health, Shanxi Medical University, Taiyuan, China; ^2^Department of Infectious Disease Control, Center for Disease Control and Prevent at Shizuishan City, Shizuishan, China; ^3^Department of Epidemiology and Statistics, School of Public Health and Management, Ningxia Medical University, Yinchuan, China

**Keywords:** primary health worker, Community Attitudes toward Mentally Ill scale, validation, reliability, stigma

## Abstract

**Objective:**

To assess the psychometric properties of the short form Community Attitudes toward Mentally Illness (SF-CAMI) scale among medical students and primary healthcare workers in China.

**Methods:**

Original English version CAMI was translated following a standard procedure. and then short-form CAMI developed through the multistage procedure. The psychometric properties were tested among two separate samples which contained 1,092 primary healthcare workers and 1,228 medical students. Reliability was assessed by internal consistency reliability and test–retest reliability. Exploratory factor and confirmatory factor analyses were performed to determine the structure and to assess the validity of the scale.

**Results:**

The Chinese version of SF-CAMI consists of 20 items and with three subscales: Benevolence, Fear and Exclusion, and Support and Tolerance. The confirmatory factor analysis indicated good fitting models for medical students and primary healthcare workers. The Cronbach *α* of total scale for both samples was good (0.82 for medical students and 0.85 for primary healthcare workers), and acceptable test–retest reliability was found (intraclass correlation coefficient is 0.62 for medical students and 0.60 for primary healthcare workers).

**Conclusion:**

The Chinese version of SF-CAMI performed good reliability and validity among both primary healthcare workers and medical students, provide more feasible and available tools for assessing the effect of mental health service programs in China.

## Introduction

Mental disorders have become the main cause of disease burden and disability in China as well as globally (1). The twelve-month prevalence of common mental disorders in adults is about 20%, and the lifetime prevalence is 29.2% (2). The mental disorders' related burden is estimated to account for 32.4% of years lived with disability (YLDs) and 13.0% of disability-adjusted life-years (DALYs) in the global burden of diseases (3). China's economic reforms have achieved great success over the past three decades; however, rapid urbanization and economic growth are generating new challenges for the country and its mental health system (4). The disease burden of mental disorders and the needs of mental health services are increasing rapidly in China (5). In the year 2009, an estimated 173 million adults have a diagnosable psychiatric disorder, of whom 158 million have never received any treatment (6). The most released data showed that the prevalence of common mental disorders is 9.3% in adult Chinese (7), and over 5.4 million patients with severe psychotic illness were receiving mental health services from the primary healthcare system due to the lack of professional psychiatry resources (8). Like all the other countries, one of the serious challenges of mental health investments is how to increase the awareness of mental health and to reduce the stigma towards mental illness (9).

In line with these needs, China has addressed the issue of stigma and mental health literacy in a national mental health law in the year 2013 (10). The National Mental Health Work Plan of China covering 2015–2020 specifies that by 2020 the level of mental health awareness in the community residents should reach 70% in urban areas and 50% in rural areas and reduce the stigma and social distance towards mental illness significantly among communities and health care professionals (11). Hence lack of well-established instruments to measure the attitudes towards mental illnesses is the bottleneck that seriously undermines any attempt to evaluate the effectiveness of public interventional programs aimed at increasing mental health literacy or decreasing stigma.

There is a need for a brief but comprehensive measure of attitude towards mental illness that can be included in the epidemiological study. As a response to this need, a blanket of instruments has been developed (12). One example is the opening minds scale for Health Care Providers (OMS-HC), a 20-item scale that assesses two dimensions of attitudes towards people with mental illness (13). Another example is the internalized stigma of mental illness (ISMI), with 29 items designed to measure the subjective experience of stigma, with subscales measuring Alienation, Stereotype Endorsement, Perceived Discrimination, Social Withdrawal, and Stigma Resistance (14). While there are numerous instruments that seek to understand specific components related to stigma, one that has been administered to the public to capture these attitudes is the Community Attitudes toward the Mentally Illness (CAMI). The scale contains 40 items with four subscales that were initially created to examine authoritarianism, benevolence, social restrictiveness, and community mental health ideology of the community members (15, 16). The CAMI has been examined in countries throughout the world and translated into different languages, including Swedish (17), Korean (18) and German (19).

The National Mental Health Work Plan of China (2015–2020) points out that a service model based on treating severe illnesses in hospitals and managing rehabilitation in communities should be established, which demands that over 50% of homebound patients with severe mental illnesses should receive community-based rehabilitation services by 2020 (11). One of the main aims of this model on the patients' rehabilitation process in the community is to reduce the stigma which was covered by the CAMI, thus we chose this scale in our study. The Chinese version of CAMI has first appeared in literature as early as 1998; the psychometric properties were tested using a psychiatry professionals sample from a psychiatric hospital in Beijing (20). However, that study did not assess test–retest reliability and failed to publish the Chinese version of CAMI in literature society. Furthermore, as a 40-item instrument, its acceptance will be limited in a large scale epidemiological study where time is always a consideration to avoid overburdening the respondents.

To our knowledge, there is not a standardized short form of CAMI that has been developed. The present study aimed to develop a short-form Chinese version of CAMI and to assess the psychometric properties of the SF-CAMI in two separate diversity samples of medical students and primary healthcare workers in China and to explore the difference of the attitude towards mental illness between the two groups.

## Methods

### The Translation and Content Validity of the CAMI

The translation followed a standard procedure (21); firstly, the cultural adopted version of CAMI was translated into Chinese by two bilingual mental health scholars separately (one is a native English speaker from Sydney University, Australia). Secondly, the two translations were compared, and discrepancies were reconciled to arrive at a common Chinese version. For the cultural adaptation, we modified some words and statements, for example, replaced “tax money” with “funding”, “mental health facilities” with “mental health institutions”, respectively. Thirdly, the draft of the Chinese version was back-translated into English by a bilingual mental health scholar. The back-translated English version was then compared to the original English version to decide whether the questions were properly translated, and discrepancies were resolved. Fourthly, the content validity was applied through administration to five mental health professionals (from two mental health centers) and thirty-five medical students. The articles measured content validity by a four point content validity index, including relevance, clarity, simplicity, and ambiguity (22). The content validity index and culture adaptation of the modified Chinese version of CAMI are acceptable.

### The Development of Short-Form CAMI Scale

The SF-CAMI was developed through multistage procedures (23). The full Chinese version of CAMI was performed to 352 medical students who selected using a clustering sampling method (24) in a medical university; of them, 297 participants completed the full questionnaire and included in the final analysis.

The total scale's Cronbach *α* was 0.714, and Cronbach *α* if items were deleted was shown in **Table 1**. Firstly, five items (items number 1, 5, 9, 23, 29) excluded from the original 40-item scale depend on the Cronbach *α* if the items deleted represent the increase or decrease in the sample value of Cronbach *α* if dispensing with a scale component (25).

**Table 1:** The summary of exploratory factor analysis and correlation test.

Item number	*α* if items deleted	CITC	Factors				Retained
			1	2	3	4	
1	**0.728**	−0.050	−0.037	−0.184	−0.084	0.660	no
2	0.711	**0.174**	0.111	0.038	0.427	−0.288	no
3	0.705	0.265	−0.097	0.644	−0.017	−0.063	yes
4	0.711	**0.171**	−0.123	0.520	0.048	−0.139	yes
5	**0.723**	0.006	−0.108	0.500	−0.094	−0.422	no
6	0.702	0.321	0.133	0.255	0.085	**0.340**	no
7	0.707	0.242	−0.221	0.405	0.212	0.124	yes
8	0.704	**0.124**	0.099	−0.016	0.247	0.544	no
9	**0.731**	−0.242	−0.297	0.143	−0.094	−0.385	no
10	0.710	**0.195**	−0.172	−0.207	0.653	0.174	yes
11	0.699	0.373	0.057	0.171	0.248	0.409	yes
12	0.702	**0.168**	0.089	0.081	0.505	0.040	no
13	0.701	0.330	−0.181	0.237	0.491	0.068	yes
14	0.697	0.462	0.400	0.009	0.247	0.227	yes
15	0.702	0.343	0.047	0.290	**0.349**	−0.101	no
16	0.710	**0.188**	−0.012	0.322	−0.149	0.397	no
17	0.714	0.298	−0.001	0.079	−0.033	0.427	yes
18	0.706	0.272	0.178	0.001	0.464	−0.216	no
19	0.705	0.282	0.225	0.491	−0.268	0.199	yes
20	0.704	0.349	0.057	−0.022	0.557	0.087	yes
21	0.698	0.407	0.039	0.008	0.644	0.075	yes
22	0.707	0.252	0.574	−0.164	0.132	0.005	yes
23	**0.729**	−0.172	0.143	−0.188	−0.219	−0.052	no
24	0.704	0.286	**0.299**	0.225	−0.087	0.265	no
25	0.697	0.462	0.554	0.044	0.237	0.065	yes
26	0.717	**0.080**	0.021	−0.079	0.306	−0.096	no
27	0.716	**0.102**	0.294	−0.043	−0.072	0.180	no
28	0.707	0.237	−0.320	**0.392**	0.264	0.153	no
29	**0.723**	−0.066	0.301	−0.218	−0.152	0.019	no
30	0.706	0.261	0.498	−0.173	0.300	−0.082	yes
31	0.703	0.327	**0.330**	0.152	0.306	−0.222	no
32	0.705	0.271	0.284	0.476	−0.248	0.107	yes
33	0.719	**0.053**	0.386	−0.038	−0.227	0.169	no
34	0.706	0.274	**0.319**	0.113	0.220	−0.185	no
35	0.715	**0.094**	0.319	−0.100	−0.012	0.133	no
36	0.704	0.295	−0.045	0.520	0.132	−0.088	yes
37	0.708	0.231	−0.004	−0.036	0.434	0.140	yes
38	0.704	0.325	0.617	0.097	0.002	−0.097	yes
39	0.712	0.371	0.089	0.081	0.505	0.040	yes
40	0.701	0.340	0.127	0.481	−0.069	0.175	yes

Bolded are exclude items.

Secondly, the corrected item-total correlations (CITC), which is the correlation of an item with the scale omitting this item, were calculated; nine more items excluded (item numbers 2, 8, 10, 12, 16, 26, 27, 33, 35) depend on the criteria that CITC larger than 0.20 is acceptable (26). One item with a corrected CITC of 0.171 was kept due to the consideration of several aspects: (i) this item had a good performance in factor analysis; (ii) current study aimed to shorten the original scale into 20 items to avoid over damage of the validation of the original scales; (iii) the item “The most effective therapy for many mental patients is to let them go back to a normal community” was identified as a very important dimension of the whole thematic framework of attitudes toward mental illness in China.

Thirdly, exploratory factor analysis (EFA) was used to explore the latent factor structure of the full CAMI. The factorability of the correlation matrix was demonstrated by an acceptable KMO (KMO = 0.72) and Bartlett's test of sphericity (χ^2^ = 2,297.08, p < 0.001). Examination of associated eigenvalues, scree plot and drawing from the factor structure of the original CAMI scales which is a four-factor model, these items revealed four latent variables. A Promax rotation was used in EFA; those items with a lower factor loading less than 0.4 were deleted (27), resulting in six more items excluded (item numbers 6, 15, 24, 28, 31, 34). As shown in **Supplementary Table 1**, the final SF-CAMI contain 20 items.

In the fourth stage, EFA was employed to identify the structure of the SF-CAMI. As shown in **Supplementary Table 2**, there are three factors identified. Factor 1 was named benevolence which plays an important role in Confucianism society. Factor 2 was named fear and exclusion; the main theme was reluctance to contact with mental illness patients intimately (*e.g.* “I would not want to have a neighbor who has been mentally ill”), their exclusion from communities (*e.g.* “Mental health facilities should be kept out of residential neighborhoods”) and fear of them (*e.g.* “It is frightening whenever to think of people with mental problems living nearby”). Factor 3 was named support and tolerance; the main theme was more support and tolerance should be taken to mental illness (*e.g.* “The situation that the mentally ill has for too long been the subject of ridicule should be put to an end”. “Residents should accept the location of mental health institutions in their neighborhood to serve the needs of the residents”).

### The Test of the Reliability of SF-CAMI

#### Participants

The reliability and validity of SF-CAMI were assessed among two separate large samples. A cluster sampling method was used to select medical students. Those who registered in the same class were defined as a cluster (usually 35–45 students). Twenty-eight classes in a medical university were selected with a total sample of 1,314 students that received the survey; of them, 1,228 (93.4%) finished the full questionnaire and were included in the data analysis. As shown in **Table 2**, the average age was 20.8 years with a standard deviation of 1.6, and 36.6% of them were male, 32.4% of them were minorities, and 30.86% of students had previously enrolled in psychiatry courses.

**Table 2:** Demographic characteristics of medical student and primary health care worker.

Characteristic		Student (*n* = 1,228)	PHW (*n* = 1,092)
Mean age, yr. (*SD*)		20.8 (1.6)	36.3 (10.2)
Gender, *n* (%)	Male	450 (36.6)	332 (30.4)
	Female	778 (63.4)	760 (69.6)
Education, yr. *n* (%)	<12	0	178 (16.3)
	12-	0	539 (49.4)
	≥15	1,228 (100.0)	375 (34.3)
Ethnicity, *n* (%)	Han	830 (67.6)	870 (79.7)
	Minority	398 (32.4)	222 (20.3)
Rural/urban, *n* (%)	Rural	695 (56.6)	498 (45.6)
	Urban	533 (43.4)	594 (54.4)
Professional, *n* (%)	TCM	*NA*	61 (5.6)
	Western Medicine	*NA*	432 (39.6)
	Nurse	*NA*	303 (27.7)
	Public Health Doctor	*NA*	126 (11.5)
	Other	*NA*	170 (15.6)

TCM, Traditional Chinese Medicine; PHW, Primary health worker; NA, Not Apply.

Primary healthcare workers were selected using a quota sampling method. Ninety-five primary healthcare centers were selected from a total of 345 center lists depending on the successful contact with the centers and the geographical distribution in the whole province. All the healthcare workers registered in the selected centers eligible enrolled in the study, resulting in a total of 1,520 potential participants; 1,200 received the survey. Finally, 1,092 (91.0%) finished the full questionnaire and were included in the data analysis. As shown in **Table 2**, the average age was 36.3 years with a standard deviation of 10.2; most of the primary healthcare workers were female and with less than twelve years of school education, 20.3% of them were minorities, and approximately 40.0% of them were physicians.

#### Procedure

The survey was conducted during a one-week period by the research team for college students; the questionnaire was administered to students in classrooms at the university and was collected at that time. For primary healthcare workers, the questionnaire was administered to them in their institutions by four trained team members from May 1^st^, 2015 to August 20^th^, 2016.

The same survey was then re-administered one week later to a random subsample of 131 (three classes) college students and 155 primary healthcare workers to determine test–retest reliability; 110 students and 102 primary health workers completed the survey a second time.

### The Statistical Analysis

EFA was performed with Promax rotation in each sample to assess the factor structure of the Chinese version of SF-CAMI. The Kaiser–Meyer–Olkin (KMO) index was computed to assess sampling adequacy, and Bartlett's test of sphericity was used to assess the factorability of the data. A scree plot of the individual factor and cumulated factor loadings was examined. Cronbach's alpha was used to assess the internal consistency, where Cronbach's alpha coefficients greater than 0.70 are considered acceptable (28). The intraclass correlation coefficient (ICC) was used to assess test–retest reliability, where ICCs between 0.41 and 0.60 indicate moderate reliability, those between 0.61 and 0.80 represent good reliability, and those higher than 0.80 indicate excellent reliability (29). The normality of the SF-CAMI was examined using the scores of skewness and kurtosis. Either the skew scores > 2 or kurtosis values > 7 were used as reference values for determining substantial non-normality (30). The item scores and total score of SF-CAMI among the two samples were compared using the Student t-test. The significance level was set at 0.05.

AMOS 17.0 software (SPSS Inc., Chicago, IL, USA) was used to conduct confirmatory factor analysis (CFA) among the two groups of respondents separately. The maximum likelihood was used to estimate the factor loadings, the variance of the latent variable was fixed at 1 (so the loadings of the observed variables can be freely estimated), and the indices used to access model fit were chi-square, comparative fit index (CFI), Tucker-Lewis index (TLI), goodness-of-fit index (GFI), adjusted goodness-of-fit index (AGFI), and root mean square error of approximation (RMSEA). The value of RMSEA less than 0.07, with CFI equal 0.92 or higher indicates good model fit (31).

## Results

### The Validity of the SF-CAMI Version

The KMO index for the two samples was 0.86 and 0.87, respectively. The Bartlett's test of sphericity indicated that both samples were factorable at p < 0.001. Principle axis factoring analysis with Promax rotation revealed a three factor structure of SF-CAMI accounting for 45.73% of the total variance explained in the medical students and accounting for 53.78% of the total variance explained in the primary healthcare workers. The rotated factor loadings were presented in **Supplementary Table 3**.

**Figure 1:**
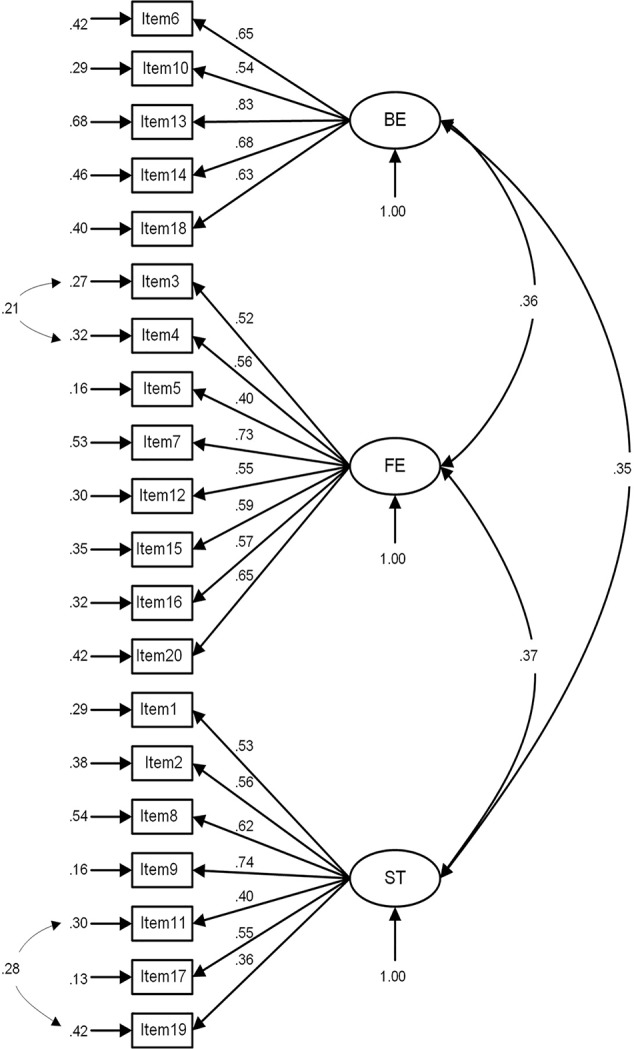
The confirmatory factor analysis of the SF-CAMI in medical students.

**Figure 2:**
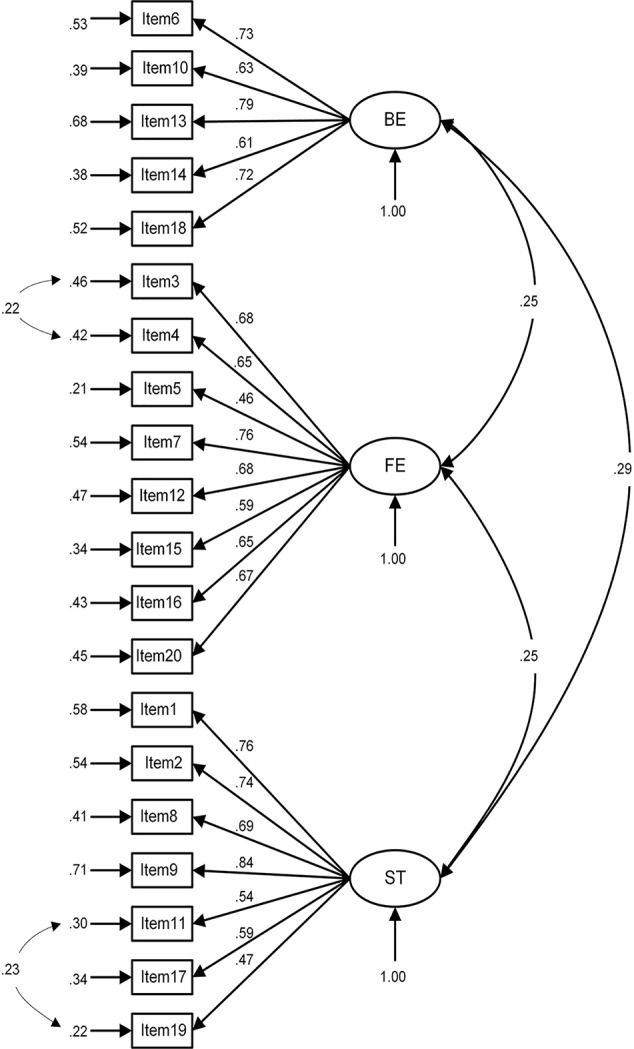
The confirmatory factor analysis of the SF-CAMI in primary healthcare workers.

The three-factor model in the medical students was shown in **Figure 1**. All standardized factor loadings were statistically significant at the 0.001 level, and the fit indices indicated a good model fit (χ^2^ = 684.60, df = 165, p < 0.001, χ^2^/df = 4.15, GFI = 0.95, CFI = 0.92, TLI = 0.90, RMSEA = 0.05, and AGFI = 0.93). For the primary healthcare workers (**Figure 2**), all of the loadings were statistically significant (p < 0.001) and two variables' loadings were below the recommended 0.50 level, and the model fit was acceptable, χ^2^ = 963.68, df = 165, p < 0.001, χ2/df = 5.84, GFI = 0.92, CFI = 0.90, TLI = 0.89, RMSEA = 0.07, and AGFI = 0.90.

Construct reliability (CR), an indicator of convergent validity, was shown in **Table 3**. The values of CR for three subscales in two samples were all above the recommended 0.70 level which suggested good convergent validity.

**Table 3:** Reliabilities and correlations between SF-CAMI subscales in medical students and primary health workers.

Factor	Student (*n* = 1,228)					PHW (*n* = 1,092)				
	M(SD)	CR	BE	FE	ST	M(SD)	CR	BE	FE	ST
BE	9.74(2.94)	0.80	0.670	*NA*	*NA*	11.26(3.78)	0.82	0.700	*NA*	*NA*
FE	21.27(4.37)	0.79	0.359*	0.578	*NA*	22.11(5.28)	0.85	0.252*	0.647	*NA*
ST	15.70(3.50)	0.74	0.354*	0.375*	0.549	15.35(4.51)	0.84	0.294*	0.248*	0.672

M, mean; SD, standard deviation; CR, construct reliability; BE, Benevolence; FE, Fear and Exclusion; ST, Support and Tolerance. *P < 0.01.

High discriminant validity is the idea that a latent construct should explain more of the variance in its item measures than it shares with another construct (31). So we compared the average variance-extracted (AVE) value of each construct with the square of the correlation estimate between any two constructs. As shown in **Table 3**, the SF-CAMI scale in two samples both had a good discriminant validity.

### The Reliability of the SF-CAMI Scale

The SF-CAMI total score and items score among the two samples were shown in **Table 4**. The primary healthcare workers had a significantly higher total score than the medical students (48.72 ± 9.46 *vs* 46.71 ± 7.81), indicating more negative attitudes towards mental illness. The Cronbach's alpha of the total scale was 0.821 for the medical students and 0.845 for the primary healthcare workers, respectively. The Cronbach's alpha values of three subscales both in the medical students (Benevolence: 0.795, Fear and Exclusion: 0.793, and Support and Tolerance: 0.744, respectively) and in the primary healthcare workers (0.825, 0.847, and 0.846 respectively) were good. As shown in **Table 4**, the corrected item-total correlations of all items were greater than 0.2, indicating good internal consistency.

**Table 4:** The Reliability of SF-CAMI Item and Scale Scores in Medical Students and Primary Healthcare Workers.

Item	*M (SD)*		Group Comparison *t*	Cohen's *d*	*α* if Item Deleted		CITC	
	Student (*n* = 1,228)	PHW (*n* = 1,092)			Student	PHW	Student	PHW
1	2.33 (0.88)	2.11 (0.89)	6.15*	0.255	0.815	0.837	0.361	0.447
2	1.82(0.77)	1.88(0.86)	−1.73	−0.074	0.815	0.837	0.366	0.466
3	2.51 (0.94)	2.54 (0.94)	−0.96	−0.040	0.814	0.835	0.398	0.494
4	2.47 (0.86)	2.62 (1.04)	−3.70*	−0.159	0.811	0.840	0.439	0.390
5	2.99(0.91)	3.24 (1.02)	−6.07*	−0.259	0.820	0.842	0.271	0.357
6	1.83 (0.77)	2.08 (0.97)	−6.63*	−0.291	0.814	0.839	0.377	0.405
7	2.47 (0.76)	2.61 (0.90)	−3.84*	−0.166	0.807	0.836	0.525	0.487
8	2.07 (0.68)	1.99 (0.78)	2.57*	0.110	0.813	0.838	0.422	0.439
9	1.93 (0.79)	2.14 (0.93)	−5.70*	−0.246	0.811	0.836	0.446	0.479
10	2.27 (0.78)	2.51 (0.96)	−6.40*	−0.279	0.819	0.842	0.270	0.340
11	2.51 (0.83)	2.52 (0.94)	−0.49	−0.021	0.816	0.838	0.349	0.420
12	2.68 (0.82)	2.89 (0.93)	−5.73*	−0.245	0.811	0.836	0.439	0.475
13	1.71 (0.75)	2.10 (0.98)	−10.72*	−0.476	0.809	0.834	0.500	0.509
14	1.84 (0.89)	2.25 (1.06)	−10.08*	−0.435	0.814	0.843	0.392	0.326
15	2.87 (0.91)	2.98 (0.95)	−2.71*	−0.113	0.811	0.839	0.443	0.411
16	2.51(0.78)	2.53(0.88)	−0.74	−0.032	0.812	0.837	0.432	0.448
17	2.20 (0.78)	2.13 (0.88)	1.94	0.081	0.816	0.838	0.338	0.440
18	2.08 (0.76)	2.32 (0.95)	−6.48*	−0.284	0.811	0.838	0.445	0.434
19	2.84(0.83)	2.58 (0.96)	7.04*	0.302	0.821	0.844	0.248	0.293
20	2.77 (0.84)	2.71 (0.95)	1.63	0.070	0.811	0.836	0.443	0.479
Total	46.71 (7.81)	48.72 (9.46)	−5.52*	−0.240	*NA*	*NA*	*NA*	*NA*

PHW, Primary Health Worker; M, mean; SD, standard deviation; CITC, Corrected Item-Total Correlation; *P < 0.05.

As shown in **Supplementary Table 4**, both the samples performed good test–retest reliability; the ICCs for the total score were 0.79 in the medical students and 0.75 in the primary healthcare workers. Most of the individual items with acceptable test–retest reliability (the ICCs ranged from 0.29 to 0.61) in the medical students, and the ICCs for individual items ranged from 0.45 to 0.74 in the primary healthcare workers.

## Discussion

The present study aimed to test the psychometric properties of a short form CAMI in two large Chinese samples and to provide a valid and reliable research tool in assessing the effect of population-based mental health programs in China or other areas where similar culture shared. The findings provide primary evidence of the acceptable psychometric properties of a short form instrument (brief but comprehensive), which can be used to assess the effects of the community mental program on the attitudes toward mental illness.

Health professionals including primary healthcare workers, nurses, psychologists, and even medical students are important targets for interventional programs of reducing stigma and social exclusion of mental illness. The current study found that both primary healthcare workers and medical students hold a somewhat negative attitude towards mental illness, consistent with previous studies out of China (32, 33), indicating that they should develop more positive, progressive, and tolerant attitudes toward people with mental illness.

As a widely-used scale, CAMI was initially developed and used in English-speaking countries, even though the Chinese version of CAMI was once used as early as in 1998. At present, the culture and the health-system environment in China had changed significantly. We half shortened the original CAMI scale within the Chinese cultural context following a standard procedure. The psychometric properties of the Chinese version of SF-CAMI in the two separate samples support the use of the scale in epidemiological study in the future.

In order to establish construct validity of the SF-CAMI scale, the study identified three dimensions (Benevolence, Fear and Exclusion, and Support and Tolerance) according to the exploratory factor analysis coupled with CFA. Good discriminant validity and CR for three subscales that demonstrated convergent validity were also given the reasonable fit of the 3-factor model; we consider the three subscales appropriate for SF-CAMI. Some previous studies also yielded a three-factor solution; for instance, Wolff et al.'s factor analysis indicated three components: Fear and Exclusion, Social Control, and Goodwill (34). Sevigny et al.'s data from physicians and nurses were analyzed through principal axis factoring and produced three factors too (20). Hogberg et al. found a Swedish version of CAMI consisting of 20 items of the original 40-item CAMI which included three-factors as Fear and Avoidance, Community Mental Health Ideology, and Open-minded and Pro-integration (17). These may demonstrate that the three-factor model is more stable across settings and culture.

Few studies assessed the construct validity of the original CAMI and modified version of CAMI using CFA. Morris et al. reported that the 20-item CAMI scale validated by Wolff was the best fit for the European nurses (35).

In this study, the fit indices of the medical students were higher than the indices of the primary healthcare workers. The possible explanations may include some differences in the structure of CAMI for these two samples the structure of the scale for the primary healthcare workers may need further exploration and modification. As shown in **Figure 1** and **Figure 2**, some variables' factor loadings below the recommended 0.5 level, such as “Mental patients need the same kind of control and discipline as a young child” and “A woman would be very unwise to marry a man who has suffered from mental illness, even though he seems to have regained normality”, have less correlation with other items from which it can be inferred that amendment to content and more analysis are needed to achieve a more statistically robust scale. Principle axis factoring analysis revealed that the SF-CAMI explained a higher proportion of the total variance than the original 40-item CAMI (53.7 *vs* 42%) (15). Also, some research has demonstrated that demographic characteristics were associated with the total scores (15, 34), so setting up a modeling for this scale requires additional variables like demographic variables which may influence scores of the SF-CAMI scale.

The test–retest reliability in the student sample was slightly weaker than that in the primary healthcare workers; three individual items had an ICC smaller than 0.40 (which was means less acceptable in reliability). The item “The situation that mentally ill have for too long been the subject of ridicule should be put to an end” had a lower ICC and was consistent with the findings revealed in factor analysis, where the item has a factor's loading much higher in the primary healthcare workers than in the medical students. It may be caused by social desirability bias (36, 37), and it is suggested that social desirability factors should be considered when using the Chinese version of SF-CAMI in the younger population.

Our results illustrated primary healthcare workers had more negative attitudes than medical students towards mental illness. A range of researches has examined associated factors including sociodemographic characteristics and experience of mental illness (38, 39). Therefore we supposed that the differences in education sustained, occupational experience, and the cultural identity between the medical students and the primary healthcare workers may contribute to it. Another possible explanation may be due to the fact that the naivety of the student population who lack work-life experience would naturally favor a more benevolent attitude than healthcare providers. Therefore, targeted integrated measures should be taken among key groups of the population to promote a more genuine attitude of “benevolence”, mitigate “fear and avoidance” and promote “support and tolerance”, which will reduce the stigma and social distancing towards mental illness.

## Limitations

As with most research, the present study has several limits. Firstly, the two samples used in this study represent subgroups that are most involved in community mental health service delivery in China. The representativeness of the samples limited the findings' generalization to whole population groups. Secondly, the instruments employed to measure the attitudes towards mental illness lacked standardized parallel measurement, leading to the failure of assessing the criterion validity of the SF-CAMI. Finally, although standard translation and back translation procedures were applied in the study by an expert team, cultural differences between China and the Western society (where the CAMI scale was originally developed and designed for use) may have affected the translation of the CAMI scale here (both the translation and the meaning of items).

## Conclusion

The Chinese version of CAMI-SF performed good reliability and validity among both primary healthcare workers and medical students in China, and the three factor model consisted of Benevolence, Fear and Exclusion, and Support and Tolerance underlying this scale. The contribution of this research is translating and developing a short adaptation and acceptable scale to assess attitude toward mental illness, which we believe can increase the feasibility and the efficiency of public mental health programs.

## Data Availability Statement

All data and materials related to the study can be obtained by contacting the corresponding author at wzhzh_lion@126.com.

## Ethics Statement

All participants provided written informed consent before completing the survey. The study was approved by the institutional review board of Ningxia Medical University (document number: 2013-167, and 2016-200)

## Author Contributions

ZW and YT participated in the design of the study. YT, SL, and YS conducted the data collection. YT conducted the statistical analysis and wrote the first draft of the manuscript. ZW oversaw the data analysis. YS checked the data and reviewed the manuscript. All the authors have read and approved the final manuscript.

## Funding

This study was funded by the China Medical Board (CMB) foundation (16-254). The funding body played no role in designing the study, collecting, analyzing, interpreting the data, writing the manuscript, or deciding to submit the paper for publication.

## Conflict of Interest

The authors declare that the research was conducted in the absence of any commercial or financial relationships that could be construed as a potential conflict of interest.

## Abbreviations

CAMI, Community Attitudes Toward the Mentally Ill; AU, Authoritarianism; BE, Benevolence; SR, Social restrictiveness; CMHI, Community mental health ideology; FE, Fear and Exclusion; ST, Support and Tolerance; KMO index, Kaiser–Meyer–Olkin index; SF-CAMI, short-form CAMI; CITC, Corrected item-total correlation; ICC, intra-class correlation coefficient; EFA, Exploration Factor Analysis; CFA, Confirmatory Factor Analysis; SEM, Structural Equation Modeling; GFI, Goodness of Fit Index; CFI, Comparative Fit Index; TLI, Tucker-Lewis Index; AGFI, Adjusted Goodness-of-Fit Index; RMSEA, Root Mean Square Error of Approximation; AVE, average variance-extracted.
