# Supplementary material for: Psychometric Properties of the Chinese Version of Short-Form Community Attitudes Toward Mentally Illness Scale in Medical Students and Primary Healthcare Workers
Source: Front Psychiatry. 2020 Apr 24;11:337. doi: 10.3389/fpsyt.2020.00337 (PMC7193688; doi:10.3389/fpsyt.2020.00337)
Supplement: Supplementary file 1 [file DataSheet_1.docx]

**Supplementary Table 1** The Chinese Version of SF-CAMI with Original English

|  | Chinese version CAMI-SF | English version CAMI-SF |
| --- | --- | --- |
| 1 | 社会上不应该过度强调有精神疾病的人对大家的危险 | There should not be any over-emphasis that the mentally ill endanger the public |
| 2 | 长期以来，把有精神疾病的人作为笑柄的情况应该杜绝 | The situation that mentally ill have for too long been the subject of ridicule should be put to an end |
| 3 | 不应该把有精神问题的人与社区中的其他人隔离 | The mentally ill should not be isolated from the rest of the community |
| 4 | 对于多数患有精神疾病的人来说，最有效的治疗就是让他们重新生活在正常人群中 | The most effective therapy for many mental patients is to let them go back to a normal community |
| 5 | 有精神疾病的人就和孩子一样需要约束与管教 | Mental patients need the same kind of control and discipline as a young child. |
| 6 | 在精神卫生服务方面增加财政支出是一种浪费 | Increased spending on mental health services is a waste |
| 7 | 有精神疾病的人并不像大多数人想象的那么危险 | The mentally ill are far less of a danger than most people imagine |
| 8 | 居民应该接受在他们的社区内设置精神卫生服务机构，以便满足居民的需要 | Residents should accept the location of mental health institutions in their neighborhood to serve the needs of the residents |
| 9 | 有精神疾病的人不应该被当成社会的遗弃者来对待 | The mentally ill should not be treated as if they are outcasts of society |
| 10 | 现有的精神卫生服务机构已经很充足了 | There have been sufficient existing facilities of mental health services |
| 11 | 一个女人和患有精神疾病的男人结婚，即使他现在已经恢复正常了，也是不明智的 | A woman would be very unwise to marry a man who has suffered from mental illness, even though he seems to have regained normality |
| 12 | 精神卫生服务机构应该设立在居民区以外 | Mental health facilities should be kept out of residential neighborhoods |
| 13 | 对待有精神疾病的人最好的措施就是把他们锁在房间里 | The best way to handle the mentally ill is to keep them behind locked doors |
| 14 | 有精神疾病的人不应该得到大家的同情 | The mentally ill don’t deserve our sympathy. |
| 15 | 我不想有一个患有精神疾病的邻居 | I would not want to have a neighbor who has been mentally ill |
| 16 | 对于有精神疾病的人来到社区里接受精神卫生服务，居民没有什么可怕的 | Residents have nothing to fear from people coming into their neighborhood to obtain mental health services |
| 17 | 实际上，任何人都有可能得精神疾病 | Virtually anyone can become mentally ill |
| 18 | 最好不要与有精神问题的人有任何来往 | It is best not to have any contact with a person who has mental problems |
| 19 | 大多数住过精神疾病医院的妇女都能照顾婴儿 | Most women who were once patients in a mental hospital can be trusted to take care of babies |
| 20 | 每当想起有精神问题的人住在附近就感到害怕 | It is frightening whenever to think of people with mental problems living nearby |

**Supplementary Table 2** The EFA of the SF-CAMI in Medical Students (n=352)

|  | Factor 1 | Factor 2 | Factor 3 |
| --- | --- | --- | --- |
| **Benevolence (Factor 1)** |  |  |  |
| Increased spending on mental health services is a waste | **0.551** | 0.050 | 0.197 |
| There have been sufficient existing facilities of mental health services | **0.594** | -0.090 | -0.092 |
| The best way to handle the mentally ill is to keep them behind locked doors | **0.698** | 0.149 | 0.069 |
| The mentally ill don’t deserve our sympathy. | **0.688** | -0.058 | -0.037 |
| It is best not to have any contact with a person who has mental problems | **0.648** | 0.214 | -0.103 |
| **Fear and Exclusion (Factor 2)** |  |  |  |
| The mentally ill should not be isolated from the rest of the community | -0.171 | **0.554** | 0.219 |
| The most effective therapy for many mental patients is to let them go back to a normal community | -0.101 | **0.512** | 0.188 |
| The mentally ill are far less of a danger than most people imagine | -0.227 | **0.432** | 0.299 |
| Mental patients need the same kind of control and discipline as a young child. | 0.073 | **0.312^a^** | -0.027 |
| I would not want to have a neighbor who has been mentally ill | 0.116 | **0.691** | -0.224 |
| Mental health facilities should be kept out of residential neighborhoods | 0.158 | **0.614** | -0.173 |
| Residents have nothing to fear from people coming into their neighborhood to obtain mental health services | -0.049 | **0.503** | 0.262 |
| It is frightening whenever to think of people with mental problems living nearby | 0.070 | **0.579** | -0.013 |
| **Support and Tolerance (Factor 3)** |  |  |  |
| The situation that mentally ill have for too long been the subject of ridicule should be put to an end | 0.100 | -0.272 | **0.605** |
| A woman would be very unwise to marry a man who has suffered from mental illness, even though he seems to have regained normality | 0.138 | 0.262 | **0.395^b^** |
| There should not be any over-emphasis that the mentally ill endanger the public | -0.053 | 0.079 | **0.629** |
| Residents should accept the location of mental health institutions in their neighborhood to serve the needs of the residents | 0.333 | -0.087 | **0.523** |
| The mentally ill should not be treated as if they are outcasts of society | 0.361 | -0.016 | **0.632** |
| Virtually anyone can become mentally ill | 0.177 | -0.098 | **0.412** |
| Most women who were once patients in a mental hospital can be trusted to take care of babies | -0.139 | 0.053 | **0.412** |

1. It is item 17 b.It is item 11

**Supplementary Table3** Rotated Factor Loadings of SF-CAMI in Two Samples

| Items | College students  n=1,228 | | | Health worker  n=1,092 | | |
| --- | --- | --- | --- | --- | --- | --- |
|  | BE | FE | ST | BE | FE | ST |
| **Benevolence** | | | |  | | |
| 6 | **0.669** | -0.027 | 0.014 | **0.741** | -0.100 | 0.096 |
| 10 | **0.605** | -0.067 | -0.050 | **0.648** | 0.011 | -0.061 |
| 13 | **0.794** | 0.042 | 0.020 | **0.758** | 0.075 | 0.043 |
| 14 | **0.644** | -0.011 | 0.052 | **0.619** | 0.022 | -0.064 |
| 18 | **0.585** | 0.122 | 0.010 | **0.702** | 0.037 | 0.024 |
| **Fear and Exclusion** | | | | | | |
| 3 | -0.113 | **0.564** | 0.087 | -0.066 | **0.660** | 0.127 |
| 4 | -0.078 | **0.573** | 0.106 | -0.134 | **0.674** | 0.033 |
| 5 | 0.131 | **0.433** | -0.191 | 0.140 | **0.444** | -0.045 |
| 7 | -0.051 | **0.715** | 0.059 | -0.066 | **0.766** | 0.018 |
| 12 | 0.161 | **0.513** | -0.061 | 0.044 | **0.678** | -0.017 |
| 15 | 0.044 | **0.607** | -0.049 | 0.134 | **0.592** | -0.113 |
| 16 | -0.015 | **0.515** | 0.102 | -0.073 | **0.651** | 0.077 |
| 20 | 0.010 | **0.666** | -0.065 | 0.129 | **0.660** | -0.073 |
| **Support and Tolerance** | | | | | | |
| 1 | -0.112 | 0.107 | **0.546** | -0.071 | 0.044 | **0.745** |
| 2 | 0.132 | -0.073 | **0.540** | 0.021 | -0.002 | **0.727** |
| 8 | 0.031 | 0.026 | **0.596** | 0.014 | -0.018 | **0.703** |
| 9 | 0.083 | -0.062 | **0.709** | -0.001 | -0.046 | **0.838** |
| 11 | 0.108 | 0.021 | **0.390** | 0.170 | -0.044 | **0.543** |
| 17 | 0.003 | -0.014 | **0.544** | 0.059 | 0.057 | **0.556** |
| 19 | -0.149 | -0.009 | **0.473** | -0.110 | 0.020 | **0.540** |

BE:Benevolence; FE:Fear and Exclusion; ST: Support and Tolerance

**Supplementary Table 4** Test-Retest Reliability of SF-CAMI Item and Scale Scores in Medical students and Primary Healthcare Workers

| Item  or  Factor | Medical Student  (n=110) | | ICC | Primary Healthcare Worker  (n=142) | | ICC |
| --- | --- | --- | --- | --- | --- | --- |
|  | Time 1  *M (SD)* | Time 2  *M (SD)* |  | Time 1  *M (SD)* | Time 2  *M (SD)* |  |
| 1 | 2.27 (0.92) | 2.45 (0.79) | 0.49 | 2.35 (1.07) | 2.24 (0.90) | 0.58 |
| 2 | 1.62 (0.72) | 1.98 (0.78) | 0.29 | 1.78 (0.89) | 1.88 (0.75) | 0.66 |
| 3 | 2.59 (0.95) | 2.48 (0.74) | 0.61 | 2.39 (1.02) | 2.41 (0.87) | 0.71 |
| 4 | 2.51 (1.02) | 2.45 (0.71) | 0.60 | 2.08 (0.86) | 2.25 (0.83) | 0.66 |
| 5 | 3.51 (0.83) | 3.48 (0.73) | 0.41 | 3.66 (0.86) | 3.43 (0.74) | 0.45 |
| 6 | 1.70 (0.70) | 1.95 (0.78) | 0.51 | 2.14 (0.88) | 2.39 (0.80) | 0.65 |
| 7 | 2.33 (0.73) | 2.46 (0.69) | 0.41 | 2.31(0.78) | 2.31 (0.77) | 0.63 |
| 8 | 1.90 (0.54) | 1.97 (0.46) | 0.60 | 1.94 (0.87) | 1.94 (0.77) | 0.69 |
| 9 | 1.85 (0.79) | 1.88 (0.57) | 0.38 | 1.87 (0.89) | 1.93 (0.74) | 0.68 |
| 10 | 2.18 (0.61) | 2.38 (0.73) | 0.32 | 2.60 (0.94) | 2.54 (0.83) | 0.62 |
| 11 | 2.53 (0.84) | 2.70 (0.83) | 0.57 | 2.90 (0.90) | 2.83 (0.93) | 0.69 |
| 12 | 3.11(0.75) | 3.06 (0.72) | 0.39 | 3.24 (1.00) | 3.17 (0.90) | 0.55 |
| 13 | 1.65 (0.64) | 1.83 (0.86) | 0.61 | 2.16 (0.77) | 2.28 (0.85) | 0.62 |
| 14 | 1.79 (0.74) | 2.07 (0.91) | 0.54 | 2.21(0.95) | 2.36 (1.01) | 0.63 |
| 15 | 3.41 (0.82) | 3.25 (0.71) | 0.60 | 3.07 (0.95) | 3.11 (0.95) | 0.60 |
| 16 | 2.63 (0.79) | 2.55 (0.71) | 0.53 | 2.57 (0.93) | 2.49 (0.85) | 0.74 |
| 17 | 2.17 (0.69) | 2.15 (0.61) | 0.49 | 2.20 (0.82) | 2.37 (0.78) | 0.67 |
| 18 | 1.93 (0.65) | 2.24 (0.80) | 0.34 | 2.36 (0.77) | 2.47 (0.81) | 0.61 |
| 19 | 1.89 (0.77) | 1.94 (0.61) | 0.54 | 3.25 (0.85) | 3.24 (0.87) | 0.48 |
| 20 | 2.77 (0.90) | 2.85 (0.85) | 0.56 | 2.82 (0.88) | 2.94 (0.92) | 0.62 |
| Total | 47.44 (6.01) | 49.13 (6.38) | 0.79 | 49.90 (6.35) | 50.60 (6.24) | 0.75 |

*M:* mean; *SD:* standard deviation; ICC: intra-class correlation coefficient;
